# On the Reaction of Carbonyl Diphosphonic Acid with Hydroxylamine and *O*-alkylhydroxylamines: Unexpected Degradation of P-C-P Bridge

**DOI:** 10.3390/molecules22071040

**Published:** 2017-06-23

**Authors:** Olga A. Khomich, Dmitry V. Yanvarev, Roman A. Novikov, Alexey B. Kornev, Elina Puljulla, Jouko Vepsäläinen, Alex R. Khomutov, Sergey N. Kochetkov

**Affiliations:** 1 Engelhardt Institute of Molecular Biology, Russian Academy of Sciences, 11991 Moscow, Russia; o.a.khomich@gmail.com (O.A.K.); JDmitry@hotmail.com (D.V.Y.); Novikovfff@bk.ru (R.A.N.); abkornev@yandex.ru (A.B.K.); alexkhom@list.ru (A.R.K); 2 School of Pharmacy, Biocenter Kuopio, University of Eastern Finland, P.O. Box 1627, FI-70211 Kuopio, Finland; elina.puljula@uef.fi (E.P.); jouko.vepsalainen@uef.fi (J.V.)

**Keywords:** Beckmann fragmentation, carbonyl diphosphonic acid, hydroxylamines

## Abstract

Derivatives of methylenediphosphonic acid possess wide spectra of biological activities and are used in enzymology as research tools as well as in practical medicine. Carbonyl diphosphonic acid is a promising starting building block for synthesis of functionally substituted methylenediphosphonates. Investigation of the interaction of carbonyl diphosphonic acid with hydroxylamine clearly demonstrates that it is impossible to isolate oxime within the pH range 2–12, while only cyanophosphonic and phosphoric acids are the products of the fast proceeding Beckmann-like fragmentation. In the case of *O*-alkylhydroxylamines, corresponding alcohols are found in the reaction mixtures in addition to cyanophosphonic and phosphoric acids. Therefore, two residues of phosphonic acid being attached to a carbonyl group provide new properties to this carbonyl group, making its oximes very unstable. This principally differs carbonyl diphosphonic acid from structurally related phosphonoglyoxalic acid and other α-ketophosphonates.

## 1. Introduction

**Figure 1:**
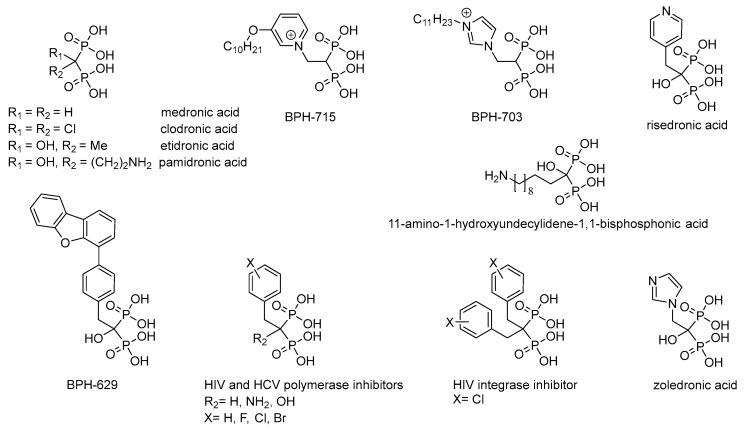
Methylenediphosphonic acid and some of its biologically active derivatives [3,4,5,6,7,9,11,12,13,14,15,16].

Non-hydrolyzable analogues of inorganic pyrophosphate, i.e., derivatives of methylenediphosphonic acid, are used in biochemistry as a source of the inhibitors of the pyrophosphate-related enzymes and for the investigation of their reaction mechanisms (for review see [1,2,3]). Medronic and etidronic acids as well as their derivatives (Figure 1), having high affinity to Ca^2+^ ions in bones, are used for treatment of osteoporosis [3]. Zoledronic and risedronic acids (Figure 1) are used to treat Paget disease [4]. Complexes of medronic and etidronic acids, and their derivatives with ^99m^Tc, are used in radiometal-based imaging of bone diseases [5]; while, for example, 11-amino-1-hydroxyundecylidene-1,1-bisphosphonic acid (Figure 1) has high practical potential as an effective chelator of heavy metals ions and rare-earth elements [6]. Besides, clodronic and pamidronic acids (Figure 1), as well as risedronic and zoledronic acids, have antitumor activity [7]. Lipophilic bisphosphonates (BPH-703 and BPH-629; Figure 1) are effective and selective inhibitors of the growth of *Plasmodium falciparum* [8] and *Mycobacterium tuberculosis* [9], that cause malaria and tuberculosis, respectively. Predominant modification of the structure of alkyl/aralkyl substituent (BPH-715, Figure 1) has resulted in powerful inhibitors of cholesterol biosynthesis, effecting both farnesyl diphosphate synthase and geranylgeranyl diphosphate synthase in nanomolar concentrations [10]. Recently, the inhibitors of reverse transcriptase [10,11] and HIV integrase [12], as well as RNA-dependent RNA polymerase of hepatitis C virus [13] have been found among the derivatives of methylenediphosphonic acids (Figure 1). It is assumed that the activity of these inhibitors is determined by effective chelation of Mg^2+^ ions in the active center of the polymerases and effective binding of aryl substituent in the hydrophobic pocket of the active enzyme center [11].

Thus, by varying the structure of the substituents at the carbon atom of methylenediphosphonic acid and the structure of chelating group, it appeared possible to change the spectrum of biological activity of bisphosphonates.

Methods of synthesis of functionally substituted methylene bisphosphonates can be divided into two main groups. The first one is comprised of the reaction of phosphorous acids derivatives with nitriles/acid chlorides/anhydrides, giving *C*-substituted methylenediphosphonic acid tetraesters with amino or hydroxyl group at the carbon of the > P-C-P < backbone [10,17,18,19]. The second group of methods proceeds from methylenediphosphonic acid tetraesters and consists in functionalization of the methylene group that is a CH-acid. Target *C*-substituted methylenediphosphonic acids are obtained from tetraesters by acidic hydrolysis [20], or using trimethylbromosilane [21]. In the case of unstable alkoxyisoprenyl derivatives, trimethylbromosilane in collidine is used [16]. However, trialkyl phosphonoglyoxalates, being structurally related to tetralkyl esters of carbonyl diphosphonic acid, are too labile to be hydrolyzed by strong aqueous acids due to the very reactive ketone function, which, unlike in simple acylphosphonates, can even undergo reaction with trimethylbromosilane [22].

**Scheme 1:**
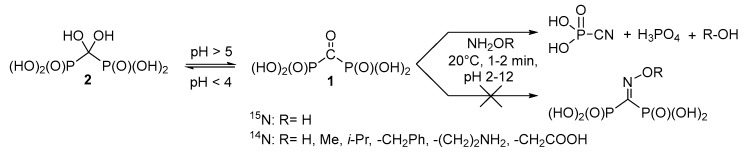
Interaction of carbonyl diphosphonic acid with hydroxylamine and *O*-alkylhydroxylamines.

Therefore, it seems reasonable to obtain a variety of functionally substituted methylenediphosphonates directly from the corresponding acids containing reactive substituents at the central carbon atom, i.e., for example, carbonyl diphosphonic acid (**1**, Scheme 1) and vinylidene diphosphonic acid. The use of the carbonyl diphosphonic acid as a starting compound seems promising for synthesis of different *O*-substituted oximes in aqueous solutions using the click chemistry reaction. Surprisingly, unsubstituted oxime and corresponding *O*-substituted oximes of carbonyl diphosphonic acid have not been a subject of systematic study yet. Thus, the goal of the present paper is to investigate the reaction of carbonyl diphosphonic acid with hydroxylamine and *O*-alkylhydroxylamines.

## 2. Results and Discussion

It is known that carbonyl diphosphonic acid **1** in aqueous solutions exists in equilibrium with dihydroxymethylenediphosphonic acid (*gem*-diol) **2**, and the equilibrium (Scheme 1) depends on the pH of the solution [23].

**Figure 2:**
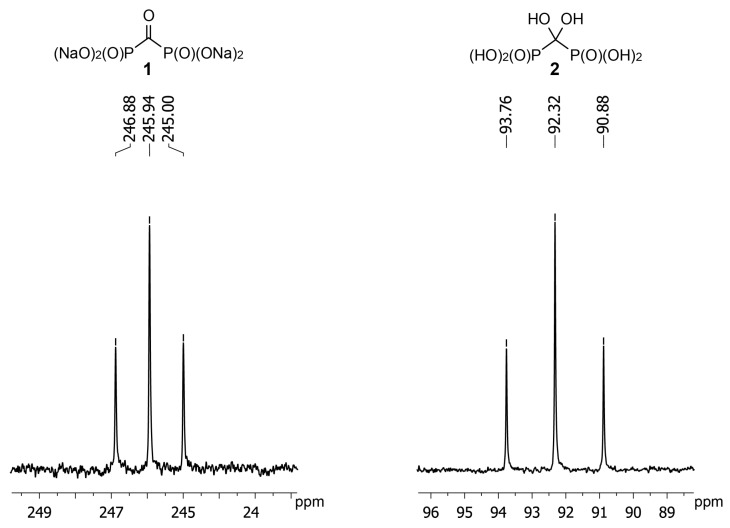
Fragments of ^13^C{^1^H}-NMR spectra of sodium carbonyl diphosphonate **1** at pH 12; δ: 245.94 (t, ^1^*J*_C-P_ 118.4 Hz) and *gem*-diol **2** at pH 3; δ: 92.32 (t, ^1^*J*_C-P_ 181.1 Hz).

Application of NMR is the most convenient method to monitor the reaction of **1** with hydroxylamine and *O*-alkylhydroxylamines. However, ^13^C-NMR data for *gem*-diol **2** are not available. We have determined that the carbon atom of *gem*-diol **2** has the chemical shift of +92 ppm (D_2_O, pH 3) (Figure 2). Having obtained crystalline acid **1** we found chemical shift of its carbon atom to be +246 ppm (D_2_O, pH 12) that is in agreement with earlier reported value of +245 ppm [24]. Such a strong shift to a weak field is quite untypical to carbonyl compounds and confirms carbonyl group of **1** to be highly electrophilic. It should also be noted that in the IR spectrum of acid **1** carbonyl stretching frequency is also observed at the unusually low value of 1612 cm^−1^ [23].

**Figure 3:**
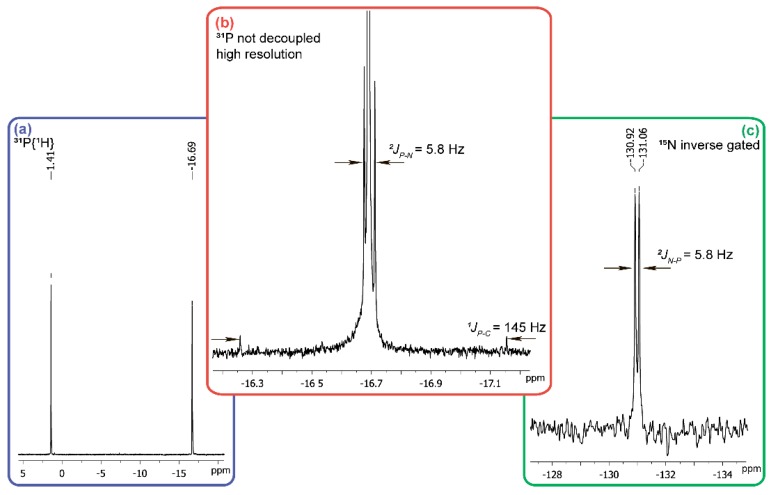
Fragments of NMR spectra of the reaction on mixing the solutions of carbonyl diphosphonic acid and ^15^NH_2_OH·HCl (each solution was adjusted to pH 12 by alkali). (**a**) ^31^P{^1^H}; (**b**) ^31^P (not decoupled, high resolution); (**c**) ^15^N–inverse gated.

It is known that neutral and alkaline aqueous solutions of acid **1** have yellow color (λ_max_ 413 nm [23]) and that also conveniently allows one to monitor transformations of **1**. The yellow color of the acid **1** solution disappears immediately on the addition of 25% molar excess of hydroxylamine both at pH 12 and at pH 6–5. In the ^31^P-NMR spectrum of the reaction mixture at pH 12 no signal of the starting acid **1** is detected, while two singlet signals at +1.4 ppm, and −16.7 ppm at a ratio of 1:1 are observed (Figure 3a). The first of them belongs to the sodium salt of phosphoric acid and introduction of sodium phosphate into the NMR tube with the reaction mixture provides an increase of the intensity of +1.4 ppm signal.

To determine the origin of the compound with the chemical shift of −16.7 ppm in ^31^P-NMR-spectrum (Figure 3a), we re-set th reaction using hydroxylamine containing 10% ^15^NH_2_OH and in this case the phosphorus atom signal in a strong field region is split into a doublet with a constant of 5.8 Hz typical for ^2^*J*_PN_ (Figure 3b). This all together with the satellites of ^1^*J*_PC_ 145 Hz (Figure 3b), being equivalent to the P-C constant in the ^13^C-NMR spectrum (δ = 126 ppm), points at the cyanophosphonate nature of the compound having the δ value of −16.7 ppm in ^31^P-NMR spectrum. The chemical shift of the nitrogen atom (δ = −130.99 ppm) of this compound (Figure 3c) is typical for the nitriles. The formation of cyanophosphonate was independently confirmed by HRMS. The signals of the compounds with *m*/*z* = 105.9698 [M − H]^−^ and *m*/*z* = 106.9659 [М − H]^−^ in the ratio of about 9:1, corresponding to ^14^N- and ^15^N-cyanophosphonate, respectively, were observed (data not shown).

The same changes in ^31^P-NMR spectra of the reaction mixtures are observed when the reaction of carbonyl diphosphonic acid with hydroxylamine is carried out at pH 2 and pH 5–6. Starting acid **1** is not detected even on mixing the reagents, just the equimolar amounts of phosphoric and cyanophosphonic acids are formed.

**Figure 4:**
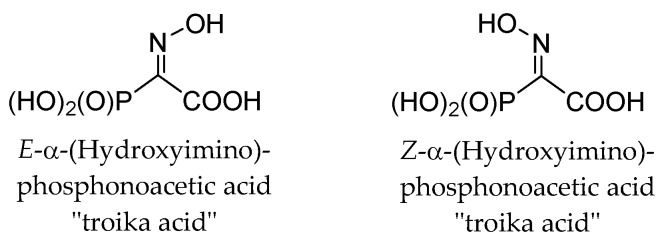
*E*- and *Z*-isomers of troika acid.

These observations are completely unexpected, since it is known that both *E*- and *Z*-isomers of structurally related α-(hydroxyimino)phosphonoacetic acid (“troika acid”) (Figure 4) are stable at alkali pH [25]. The ionization of oxime HO-group, having pKa > 10, restricted the fragmentation of troika acid since the ionized oxime oxygen can hardly function as a leaving group. Moreover, *E*- and *Z*-isomers of troika acid are obtained by alkaline hydrolysis of the corresponding *C*-monomethyl esters (*Z*-isomer is hydrolyzed more than 50 times faster than *E*-isomer [25]). However, *E*-troika acid is a mild phosphorylating agent at neutral and slightly acidic pH. The cleavage of the P-C bond (τ½ < 10 min) is likely to result in the formation of the *meta*-phosphoric acid, which has been frequently postulated to exist as a highly reactive intermediate in dissociative phosphorylation mechanisms. When the reaction is carried out in alcohols, mono-alkyl phosphates are formed in high yields [22,25]. On the contrary, at neutral or slightly acidic pH, *Z*-troika acid undergoes the Beckmann-like fragmentation (reported τ½ ~ 15 min) with the formation of cyanophosphonate [25]. Hence, these transformations proceed stereospecifically and the position of the oxime hydroxyl group in troika acid mediates phosphorylation vs. nitrile formation [25].

**Figure 5:**
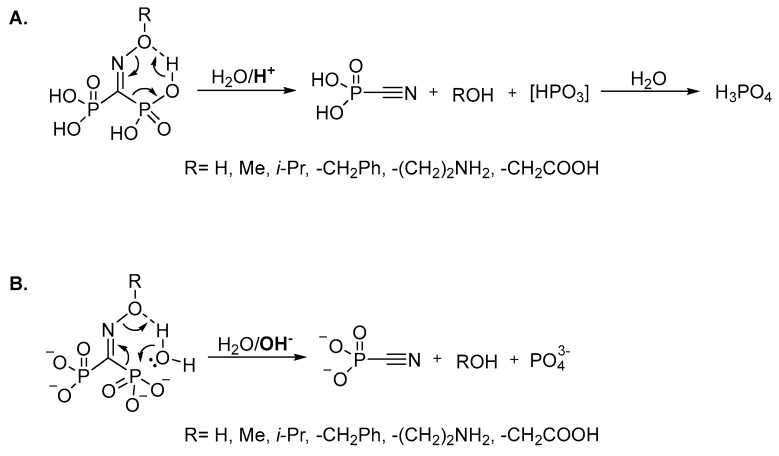
Presumed mechanism of the Beckmann-like fragmentation leading to the degradation of P-C-P bridge under acidic (**A**) and basic conditions (**B**).

It should be specially pointed out that, unlike the structurally-related troika acid, the oxime of carbonyl diphosphonic acid is unstable not only at acidic or slightly acidic pH, but also at alkaline pH due to the fast (τ½ < 1 min) Beckmann-like fragmentation leading to cyanophosphonic acid, or its salts. We assume that oxime and *O*-substituted oximes of carbonyl diphosphonic acid undergo the Beckmann-like fragmentation via six-membered intermediate (Figure 5). In acidic and neutral aqueous solutions in addition to cyanophosphonic acid, a reactive metaphosphate intermediate may be formed (Figure 5A), while at alkali pH the Beckmann-like fragmentation directly leads to the salts of cyanophosphonic and phosphoric acids (Figure 5B).

Therefore, the substitution of the carboxyl group of troika acid to the phosphonic one dramatically changes its chemical properties and the oxime of carbonyl diphosphonic acid immediately rearranges to cyanophosphonic acid even at alkali pH.

It is known that stable *O*-substituted oximes of acyl phosphonic acids are smoothly obtained by the reaction of α-ketophosphonates with *O*-alkylhydroxylamines and possess chemical properties typical for oximes. *O*-Methyl oxime of benzoylphosphonic acid can be considered an interesting exception. It is stable at acidic and neutral pH at 20 °C, while in aqueous alkali at 20 °C it very slowly (days) decomposes to phosphoric acid and benzonitrile, in contrast to the non-substituted oxime, which is stable under these conditions [26].

*O*-substituted oximes of acid **1** are not described in literature. However, carbonyl diphosphonic esters have been reported to react smoothly with H_2_NOCH_3_ forming *O*-methyl oxime of tetraisopropyl carbonyl diphosphonate at a 60% yield [22]. At the same time, chemical properties of this *O*-methyl oxime, as well as possibilities of its transformation into *O*-methyl oxime of acid 1, are have not been studied at all. Therefore, *O*-methyl hydroxylamine, branched-chain *O*-isopropyl hydroxylamine, *O*-benzyl hydroxylamine, aminooxyacetic acid, and 1-aminooxy-2-aminoethane were studied in the reaction with acid **1** (Scheme 1). 1-Aminooxy-2-aminoethane was expected to have the accelerated rate of oxime formation, since Schiff bases are formed faster than oximes and are much more reactive towards hydroxylamines, hydrazines, and semicarbazones, if compared with free carbonyl group [27,28]. The signal of starting carbonyl diphosphonic acid could not be detected in ^31^P-NMR spectra, even immediately, on mixing of carbonyl diphosphonic acid with any of tested *O*-substituted hydroxylamines at 20 °C and pH 2–12. The reaction products are sodium salts of phosphoric acid (s, +1.4 ppm) and cyanophosphonic acid (s, −16.7 ppm)—the same as in the case of hydroxylamine itself. The formation of cyanophosphonic acid was independently confirmed by HRMS. ^1^H-NMR spectra of the reaction mixtures contain signal(s) of the alcohols derived from the corresponding *O*-alkylhydroxylamines as well as the signal(s) of *O*-substituted hydroxylamines that are used in these reactions in a 25% molar excess. Respectively, short-living *O*-substituted oximes undergo the Beckmann-like fragmentation at a wide range of pH, while alkoxy groups serve as leaving groups.

Therefore, two residues of phosphonic acid attached to carbonyl group provide a unique set of properties, making it impossible to synthesize oximes of carbonyl diphosphonic acid via treatment with hydroxylamine itself and even with *O*-substituted hydroxylamines. In all cases, at 20 °C and within pH 2–12, oximes undergo the Beckmann-like fragmentation leading to cyanophosphonic acid and inorganic phosphate. This common feature of oxime and *O*-substituted oximes of acid **1** principally differs them from the structurally relative troika acid and oximes of α-ketophosphonates.

## 3. Materials and Methods

^15^N-Enriched hydroxylamine hydrochloride was prepared by the reduction of ^15^N sodium nitrite (10 atom % ^15^N) with sodium metabisulfite. Crude ^15^NH_2_OH∙HCl was purified after its conversion to acetone oxime, and the subsequent acidic hydrolysis provided ^15^NH_2_OH∙HCl with the overall yield of 65%. 1-Aminooxy-2-aminoethane dihydrochloride was synthesized as described in [29], while hydrochloride of *O*-isopropyl hydroxylamine was synthesized as described in [30]. All other reagents were purchased from Sigma-Aldrich (Steinheim, Germany) or Acros Organics (Geel, Belgium).

NMR spectra were recorded on 500.1 MHz (^1^H), 202.5 MHz (^31^P), 125.8 MHz (^13^C), and 50.8 MHz (^15^N) Bruker Avance 500 DRX (Bruker, Karlsruhe, Germany) spectrometer. Chemical shifts are reported in parts per million (ppm) using tetramethylsilane (^1^H), *tert*-butanol-d_1_ (^13^C) as internal standards, P(OMe)_3_ in CDCl_3_ (^31^P, δ = 139.8 ppm) [31], and CH_3_NO_2_ + 10% CDCl_3_ (^15^N) as external standards. ^13^C- and ^31^P-NMR spectra were proton-decoupled unless otherwise specified. High-resolution electrospray mass spectra were obtained using Applied Biosystems/MDS Sciex QSTAR XL (USA). The measurements were acquired in a negative ion mode with the following parameters: interface capillary voltage 4700 V; mass range from *m*/*z* 50 to 3000; nebulizer pressure 0.4 Bar; flow rate 3 µL/min; nitrogen was applied as a dry gas (4 L/min); interface temperature was set at 190 °C. VWR Scientific pH-meter (model 2000 with EW-5991-61-electrode, “Cole Parmer” (Vernon Hills, IL, USA) was used to perform pH measurements.

### 3.1. Synthesis of Tetraethyl 1,1-Dichloromethylene Bisphosphonate

To an ice-cold 13% water solution of NaClO (20 mL) 37% HCl (0.5 mL) was added and the pH of the resulting mixture was adjusted to 9–10 with Na_2_CO_3_ following with the addition of 4 g (13.9 mmol) of tetraethyl methylenebisphosphonate. Heterogeneous reaction mixture was stirred for 2 h at 20 °C, then DCM (20 mL) was added, organic phase was separated, washed with brine, and dried over Na_2_SO_4_ to afford 2.78 g (56%) of tetraethyl 1,1-dichloromethylene bisphosphonate (b.p. 160–166 °C/0.1 mm Hg; lit.: 119–120 °C/0.05 mm Hg [32]). ^13^C{^1^H}-NMR (CDCl_3_): δ 72.84 (t, ^1^*J*_C-P_ 151.2 Hz), 66.46 (m), 16.76 (t, ^3^*J*_C-P_ 3.1 Hz). ^31^P{^1^H}-NMR (CDCl_3_): δ 8.95 (s).

### 3.2. Synthesis of Carbonyl Diphosphonic Acid *(**1**)*

A mixture of 0.7 g (2 mmol) of 1,1-dichloromethylene bisphosphonate in 20% HCl (10 mL) was refluxed for 3 h and then concentrated in vacuo. The residue was co-evaporated with water (3 × 10 mL) and then refluxed in 2 M NaOH (6 mL) for 4 h. Yellow solution was concentrated in vacuo to 3 ml and left at 4 °C for 24 h. The precipitate was recrystallized from water to give tetrasodium salt of **1** (378 mg, 68%). ^13^C{^1^H}-NMR (D_2_O): δ 245.94 (t, ^1^*J*_C-P_ 118.4 Hz). ^31^P{^1^H}-NMR (D_2_O): δ 0.3 (s).

### 3.3. Reaction of Carbonyl Diphosphonic Acid with Hydroxylamine and Its Esters, General Procedure

**Reaction at pH 12:** To a yellow solution of 10 mg (0.036 mmol) of tetrasodium salt of **1** in D_2_O (0.3 mL) a solution of hydroxylamine hydrochloride or hydrochloride of *O*-alkylhydroxylamine (1.25 eq) in water (0.3 mL, pH was adjusted to 12) was added. Reaction mixtures became colorless on mixing the reagents and NMR spectra were registered. All transformations proceed according to NMR with quantitative yields.

With ^15^NH_2_OH: ^13^C{^1^H}-NMR (D_2_O): δ 126.0 (d, ^1^*J_P-C_* 145.0 Hz). ^31^P{^1^H}-NMR (D_2_O): δ 1.4 (s), −16.7 (d, ^2^*J*_P-N_ 5.8 Hz). ^15^N{^1^H} inverse gated NMR (D_2_O): δ −131 (d, ^2^*J*_N-P_ 5.8 Hz). HRMS (ESI): calcd. for CH_2_^14^NO_3_P [M − H]^−^: 105.9694; found: 105.9698. Calcd. for CH_2_^15^NO_3_P [M − H]^−^: 106.9664; found: 106.9659.

With NH_2_OMe: ^1^H-NMR (D_2_O): δ 3.60 (s, 3H, NH_2_OCH_3_), 3.31 (s, 3H, HOCH_3_). ^13^C{^1^H}-NMR (D_2_O): δ 126.0 (d, ^1^*J*_P-C_ 145.0 Hz), 61.3, 47.8. ^31^P{^1^H}-NMR (D_2_O): δ 1.4 (s), −16.7 (s).

With NH_2_O-*i*-Pr: ^1^H-NMR (D_2_O): δ 4.35 (sept., 1H, NH_2_OCH, ^3^*J*_H-H_ 6.1 Hz), 4.08 (sept., 1H, HOCH, ^3^*J*_H-H_ 6.2 Hz), 1.35 (d, 6H, NH_2_OCH(CH_3_)_2_, ^3^*J*_H-H_ 6.1 Hz), 1.24 (d, 6H, HOCH(CH_3_)_2_, ^3^*J*_H-H_ 6.2 Hz). ^13^C{^1^H}-NMR (D_2_O): δ 126.0 (d, ^1^*J_P-C_* 145 Hz), 81.3, 67.1, 26.6, 22.5. ^31^P{^1^H}-NMR (D_2_O): δ 1.4 (s), −16.7 (s).

With NH_2_OBn: ^1^H-NMR (D_2_O): δ 7.57 (s, 5H, NH_2_O-CH_2_C_6_H_5_), 7.52–7.50 (m, 5H, HOCH_2_C_6_H_5_), 5.07 (s, 2H, NH_2_OCH_2_), 4.72 (s, 2H, HOCH_2_). ^13^C{^1^H}-NMR (D_2_O): δ 143.2, 136.7, 132.5, 132.3, 132.0, 131.7, 130.8, 130.5, 126.0 (d, ^1^*J*_P-C_ 145 Hz), 80.2, 66.9. ^31^P{^1^H}-NMR (D_2_O): δ 1.4 (s), −16.7 (s).

With NH_2_O(CH_2_)_2_NH_2_: ^1^H-NMR (D_2_O): δ 4.18 (t, 2H, NH_2_OCH_2_, ^3^*J*_H-H_ 4.7 Hz), 3.90 (t, 2H, HOCH_2_, ^3^*J*_H-H_ 5.1 Hz), 3.38 (t, 2H, NH_2_OCH_2_CH_2_NH_2_, ^3^*J*_H-H_ 4.7 Hz), 3.22 (t, 2H, HOCH_2_CH_2_NH_2_, ^3^*J*_H-H_ 5.06 Hz). ^13^C{^1^H}-NMR (D_2_O): δ 126.0 (d, ^1^*J*_P-C_ 145.0 Hz), 73.8, 60.6, 44.3, 41.0. ^31^P{^1^H}-NMR (D_2_O): δ 1.4 (s), −16.7 (s).

With NH_2_OCH_2_COOH: ^1^H-NMR (D_2_O): δ 4.48 (s, 2H, NH_2_OCH_2_), 4.20 (s, 2H, HOCH_2_). ^13^C{^1^H}-NMR (D_2_O): δ 180.9, 178.0, 126.0 (d, ^1^*J*_P-C_ 145 Hz), 75.2, 63.4. ^31^P{^1^H}-NMR (D_2_O): δ 1.4 (s), −16.7 (s).

**Reaction at pH 2:** To a solution of 12 mg (0.043 mmol) of tetrasodium salt of **1** in 10% citric acid (0.5 mL), a solution of hydroxylamine hydrochloride or *O*-substituted hydroxylamine hydrochlorides (1.25 eq) in D_2_O (0.1 mL) was added and ^31^P NMR spectra were registered on mixing the reagents (pH of the reaction mixtures was ~2.0) demonstrating the formation of inorganic phosphate and cyanophosphonic acid with quantitative yields similar to that described above for the reaction performed at pH 12.

**Reaction at pH 5–6:** To a solution of 10 mg (0.036 mmol) of tetrasodium salt of **1** in water (0.5 mL), a solution of hydroxylamine hydrochloride or hydrochloride of *O*-alkylhydroxylamine (1.25 eq) in D_2_O (0.1 mL) was added and ^31^P-NMR spectra were registered on mixing the reagents (pH of the reaction mixtures were ~5–6, depending on *O*-substituted hydroxylamine structure) demonstrating the formation of inorganic phosphate and cyanophosphonic acid with quantitative yields similar to that described above for the reaction performed at pH 12.
